# Evidence-based new service package vs. routine service package for smoking cessation to prevent high risk patients from cardiovascular diseases (CVD): study protocol for randomized controlled trial

**DOI:** 10.1186/1745-6215-14-419

**Published:** 2013-12-05

**Authors:** Myo Nyein Aung, Motoyuki Yuasa, Thaworn Lorga, Saiyud Moolphate, Hiroshi Fukuda, Tsutomu Kitajima, Hirohide Yokokawa, Kazuo Minematsu, Susumu Tanimura, Yoshimune Hiratsuka, Koichi Ono, Prissana Naunboonruang, Payom Thinuan, Sachio Kawai, Yaoyanee Suya, Somboon Chumvicharana, Eiji Marui

**Affiliations:** 1 Department of Public Health, Juntendo University School of Medicine, Hongo 2-1-1, Bunkyo-ku, Tokyo 113-8421, Japan; 2 Boromrajonani College of Nursing Nakhon Lampang (BCNLP), Lampang, Thailand; 3 Department of Public Health, Faculty of Science and Technology, Chiang Mai Rajabhat University, Chiang Mai, Thailand; 4 Department of General Medicine, Juntendo University School of Medicine, Tokyo, Japan; 5 Graduate School of International Cooperation Studies, Kyorin University, Hachioji, Japan; 6 Department of Public Health, Hyogo College of Medicine, Nishinomiya, Japan; 7 Department of Health and Welfare Services, National Institute of Public Health, Wako, Japan; 8 Department of Ophthalmology, Juntendo University School of Medicine, Tokyo, Japan; 9 Department of Sport Medicine, Juntendo University School of Health and Sport Science, Inba, Japan; 10 Maetha Hospital, Lampang, Thailand; 11 Department of Human Arts Sciences, University of Human Arts and Sciences, Saitama, Japan

**Keywords:** Smoking, Smokerlyzer, Family, Thailand, Lampang, CVD, Primary care, Tobacco, ESCAPE trial

## Abstract

**Background:**

Smoking cessation is a high-priority intervention to prevent CVD events and deaths in developing countries. While several interventions to stop smoking have been proved successful, the question of how to increase their effectiveness and practicality in developing countries remains. In this study, a newly devised evidence-based smoking cessation service package will be compared with the existing service in a randomized controlled trial within the community setting of Thailand.

**Method/Design:**

This randomized control trial will recruit 440 current smokers at CVD risk because of being diabetic and/or hypertensive. Informed, consented participants will be randomly allocated into the new service-package arm and the routine service arm. The study will take place in the non-communicable disease clinics of the Maetha District Hospital, Lampang, northern Thailand. The new smoking-cessation service-package comprises (1) regular patient motivation and coaching from the same primary care nurse over a 3-month period; (2) monthly application of piCO + smokerlyzer to sustain motivation of smoker’s quitting attempt and provide positive feedback over a 3-month period; (3) assistance by an assigned family member; (4) nicotine replacement chewing gum to relieve withdrawal symptoms. This new service will be compared with the traditional routine service comprising the 5A approach in a 1-year follow-up. Participants who consent to participate in the study but refuse to attempt quitting smoking will be allocated to the non-randomized arm, where they will be just followed up and monitored. Primary outcome of the study is smoking cessation rate at 1-year follow-up proven by breath analysis measuring carbomonoxide in parts per million in expired air. Secondary outcomes are smoking cessation rate at the 6-month follow-up, blood pressure and heart rate, CVD risk according to the Framingham general cardiovascular risk score, CVD events and deaths at the 12-month follow-up, and the cost-effectiveness of the health service packages. Intention-to-treat analysis will be followed. Factors influencing smoking cessation will be analyzed by the structure equation model.

**Discussion:**

This multicomponent intervention, accessible at primary healthcare clinics, and focusing on the individual as well as the family and social environment, is unique and expected to work effectively.

**Trial registration:**

Current Controlled Trials ISRCTN89315117

## Background

Cardiovascular diseases (CVDs) are the leading cause of morbidity and mortality globally [1]. Developing countries such as Thailand are severely affected, and the current trend is expected to get worse over the next few decades [2,3]. Reducing the CVD burden via lifestyle modification has, therefore, become an urgent public health priority [1]. There are a number of modifiable risk factors of CVDs such as smoking, physical inactivity and unhealthy diet [4], and smoking cessation is considered to have the greatest potential to prevent thousands of CVD events and deaths [4].

Thailand is a middle-income developing country with a current smoking rate of approximately 50% among males and 5-6% among females [5,6]. The smoking rate is heterogeneous across the country and could be as high as 70% among both males and females in rural areas of northern Thailand [7]. The increasing wave of CVDs in recent years is seen as the primary cause of hospitalization and rising numbers of CVD deaths within all provinces of Thailand [8]. Thailand is a country with a strict tobacco control and an effective online service for smokers attempting to give up smoking [9,10]. However, previous surveys of tobacco use in Thailand have revealed unique features of its smokers, such as the high smoking rate in rural areas and high prevalence of “roll-your-own” cigarettes and household smoking [10]. A smoking cessation service reaching to the primary health care is key to prevention of CVD in Thailand. Enhancing the implementation of community-based smoking cessation programs would avert the health and economic burden of CVDs [10,11].

A randomized controlled trial, seeking an effective smoking cessation service in the primary care setting of developing countries, is very rare and has yet to be conducted in Thailand [12]. While smoking cessation interventions, such as brief counseling, nicotine replacement and therapy, etc., were reported to be effective, the question of how to increase their effectiveness and practicality remains [11,13]. To function as part of primary health care, a culturally-tailored smoking cessation service, which is feasible within the individual, social and financial capitals of the target setting, is a necessity. In this study, a newly devised evidence-based smoking cessation service package will be compared, in a randomized controlled trial, with the existing service within the community setting of northern Thailand.

## Methods/Design

### Study hypothesis

A smoking cessation service package comprising continuous motivation by a single designated nurse, application of a smokerlyzer, continuous family support and nicotine replacement therapy, based on individual need, will lead to a more successful behavioral change in patients at risk from cardiovascular diseases, as compared to the conventional smoking-cessation service [14].

### Study objectives

The primary objective is to compare the smoking cessation rate between a new evidence-based smoking cessation service package and the conventional 5 A approach to smoking cessation at the 1-year follow-up.

 The secondary objectives are:

1. to compare the smoking cessation rate at the 6-month follow-up.

2. to compare the CVD risk by Framingham general cardiovascular risk score among intervention and control groups at the 1-year follow-up.

3. to determine the characteristics of those who have successfully given up smoking and the determinants of behavioral change.

4. to determine the characteristics of relapse smokers after 6 months of cessation and the determinants of behavioral change.

5. to compare the cardiovascular disease events between ex-smokers, reduced smokers and continuous smokers at the 1-year follow-up.

6. to determine the cost-effectiveness of the new smoking cessation service package.

### Study design

The study design is essentially that of a randomized controlled trial (RCT) comparing two parallel groups. However, given the nature of smoking cessation research, an additional, third arm will be included for smokers who refuse to try to give up smoking and so are unable to be randomized into either of the treatment service arms. Consequently, it will be a randomized, controlled trial with an additional non-randomized arm. The study flow chart is as follows.

### Study site

Thailand is the study site. Maetha Hospital (MH) is the leading center in this trial. The study will be implemented via the MH network of mobile NCD clinics at seven primary care units (PCU) within the Maetha district. Study site PCUs were chosen based on the eligibility criteria of (1) the presence a regular mobile NCD clinic; (2) a smoking rate among NCD clinic attendants not less than 10%; (3) willingness of the PCU to join the study.

### Study population

The study population will comprise smokers with a high CVD risk, having either hypertension or diabetes, or both.

### Eligibility criteria for individual patients to enroll in the study

The selection of participants will be based on the following inclusion and exclusion criteria:

### Inclusion criteria

1. Current smokers with diabetes.

2. Current smokers with hypertension.

3. Current smokers with both diabetes and hypertension.

4. Smokers who have never succeeded in giving up smoking.

5. Male or female.

6. Aged 35–80 years.

### Exclusion criteria

1. Any female patients who are pregnant or planning to become pregnant.

2. Patients aged younger than 35 years.

3. Patients with documented type I diabetes.

4. Patients with cancer.

5. Patients with severe chronic pulmonary diseases using home oxygen therapy.

6. Patients with a known diagnosis of a previous cardiovascular disease (CVD) event.

### Ethical approval

Ethical approval of the study protocol was granted by the Juntendo University Ethics Committee, Japan, by permission no. 2012194, and the Institutional Review Board at Boromarajonani College of Nursing, Lampang, Thailand, by approval number E2556/005. The study has been registered as an international current control trial at the ISRCTN registry. The ISRCTN registration no. ISRCTN89315117 was assigned on 9 July 2013 [14] Acronym of the study is ESCAPE: *Effective Smoking Cessation Augmented PackagE*.

Written informed consent will be requested from eligible individuals. Participants can freely decide to participate in the trial after being informed about the study (Autonomy). There are no racial or ethnical criteria for inclusion in the study (Equity). Participants in each arm of the study will receive routine health services, and those in the new-service intervention arm will receive some trial-related service benefits, such as breath analysis by smokerlyzer and provision of nicotine replacement therapy if suffering from nicotine withdrawal. There will be no or minimal introduction of risk to the participants but instead an expected CVD risk reduction via the smoking cessation intervention and study-related health education (Beneficence).

### Enrollment and randomization

**Figure 1:**
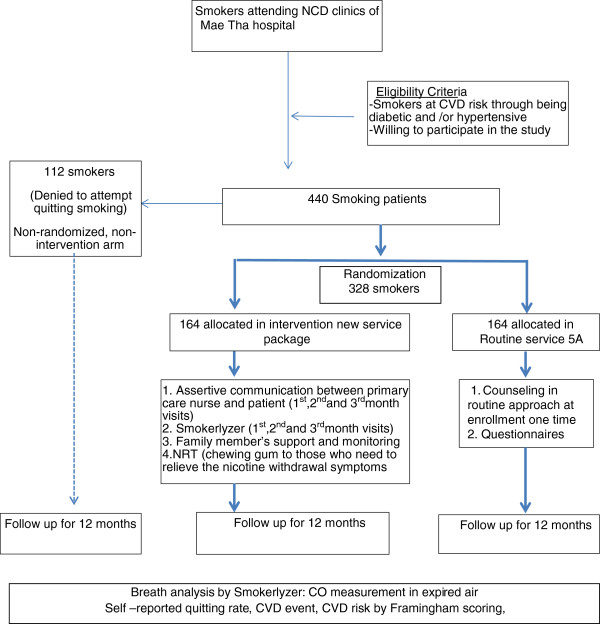
**Flow chart for enrollment and follow-up plan for RCT with an additional non-randomized arm.** Note: *Fagerstrom nicotine dependence test, *NRT* nicotine replacement therapy. *Bold arrows* show the randomized trial, and the *dotted arrow* shows the non-randomized arm.

To ensure the required sample size, smoking prevalence among diabetes and hypertension clinics attendants was surveyed. The study site is within the community setting and coupling with non-communicable disease (NCD) clinics, which is expected to promote the smoker’s access to enrollment in the study. Those meeting eligibility criteria will be invited to participate in the study in their routine visit to the PCU. The participants who consent and decide to attempt to stop smoking will be randomly allocated into either the routine service intervention arm or the new service intervention arm. Those who refuse to attempt quitting smoking will be recruited to the non-randomized arm (Figure 1). The randomization site is the NCD clinic at the PCU. Randomization is blocked with the center as a stratum. Random sequences are generated by the statistician on the basis of blocks of 24. The allocated arm for each participant will be provided to the study sites PCUs in opaque, sealed envelopes.

### Concealment of allocation

Requesting of consent and randomization will precede explanations to patients about the procedures within their allocated study arm. The allocated arm will be revealed to the patient when the research nurse opens the sealed envelope following recruitment, consent and the participant’s agreement to attempt to give up smoking. Participants will know the details only of the study arm to which he/she has been randomly allocated. The investigators cannot anticipate into which intervention arm a participant will be allocated.

### Blinding

Double blinding is not possible because of the nature of trials for health care service intervention. The PCU nurse will know the intervention arms. However, possible investigator’s bias will be minimized, and anticipation of the allocated arm will be prevented by the block randomization procedure [15].

### Intervention

An evidence-based smoking cessation intervention package was devised in order to represent a practical and replicable smoking cessation service in the primary health care setting of a developing country. The other arm of the randomized control trial represents the conventional smoking cessation service used in Thailand (Table 1).

**Table 1:** Timetable of interventions in the randomized controlled trial and additional non-randomized arm of the ESCAPE study

		**Intervention**											
Visit number	1	2	3	4	5	6	7	8	9	10	11	12	13
Month	0	1	2	3	4	5	6	7	8	9	10	11	12
**New package service**													
Assertive communication	x	x	x	x									
Nicotine replacement chewing gum to those indicating need	±	±	±	±	±	±	±						
Family member assistance	x	x	x	x	x	x	x	x	x	x	x	x	x
Smokerlyzer as motivational tool		x	x	x									
**Existing service**													
Health care worker counseling patient at enrollment	x												
Routine 5 A service including assessment of nicotine dependence questionnaires	x												
Advice and encouragement on NCD clinic follow-up visits	x						x						x
**Refusing to give up smoking**													
Recruitment	x												

± shows the intervention to those who have indicated a need for nicotine replacement therapy.

#### New evidence-based service (intervention arm)

The new evidence-based service package arm will comprise the following interventions [14]: (1) during the first meeting, a primary care unit (PCU) nurse will motivate the patient to give up smoking by providing a clear explanation, and the same nurse will provide repeated advice each month for the next 3 months; (2) a piCO ^+^ smokerlyzer will be used to show the level of carbon monoxide (CO) the patient breathes out and the improvement of the patient’s lung health over 3 successive months; (3) a PCU nurse will train one member of the patient’s family on how to care for the patient until he/she has successfully given up smoking; (4) patients who suffer from nicotine craving are given nicotine replacement chewing gum.

#### The existing service arm (control arm of the randomized control trial)

The routine health service smoking cessation package will include the following: (1) during the first meeting the hospital healthcare worker advises the patient on how to stop smoking; (2) the patient answers questions about their smoking habit to indicate their level of nicotine dependency; (3) the patient is reminded by the healthcare worker on subsequent visits to the hospital how to stop smoking; (4) the patient is asked to let the healthcare worker know if and when he/she has successfully been able to give up smoking.

#### Difference between two service arms

The two service arms differ in several components (Table 1). In the new service arm, nursing intervention is an assertive communication between a patient and nurse to achieve the goal of stopping smoking rather than conventional patient counseling by the nurse or any health care staff as would be the case for routine arm group [16]. The PCU nurse will ask the patient what he/she knows about the risks of smoking and will provide education about them. Then an assertive form of communication will be established between the nurse and patient assisting in the patient’s attempt to give up smoking. The same PCU nurse will sustain such communication, along with the implementation of an action plan, for a period of 3 months after enrollment. This communication may include phone calls to the participant and his/her family.

Second, participants in the new service arm group will be able to see both the level of their nicotine dependence, via the colored diode light in the smokerlyzer breath analysis, and also the level of CO in their expired air, as compared to their questionnaire rating as would be the case for the routine arm group. This will provide both visible positive feedback and an achievable goal in the assertive communication between the nurse and patient.

**Figure 2:**
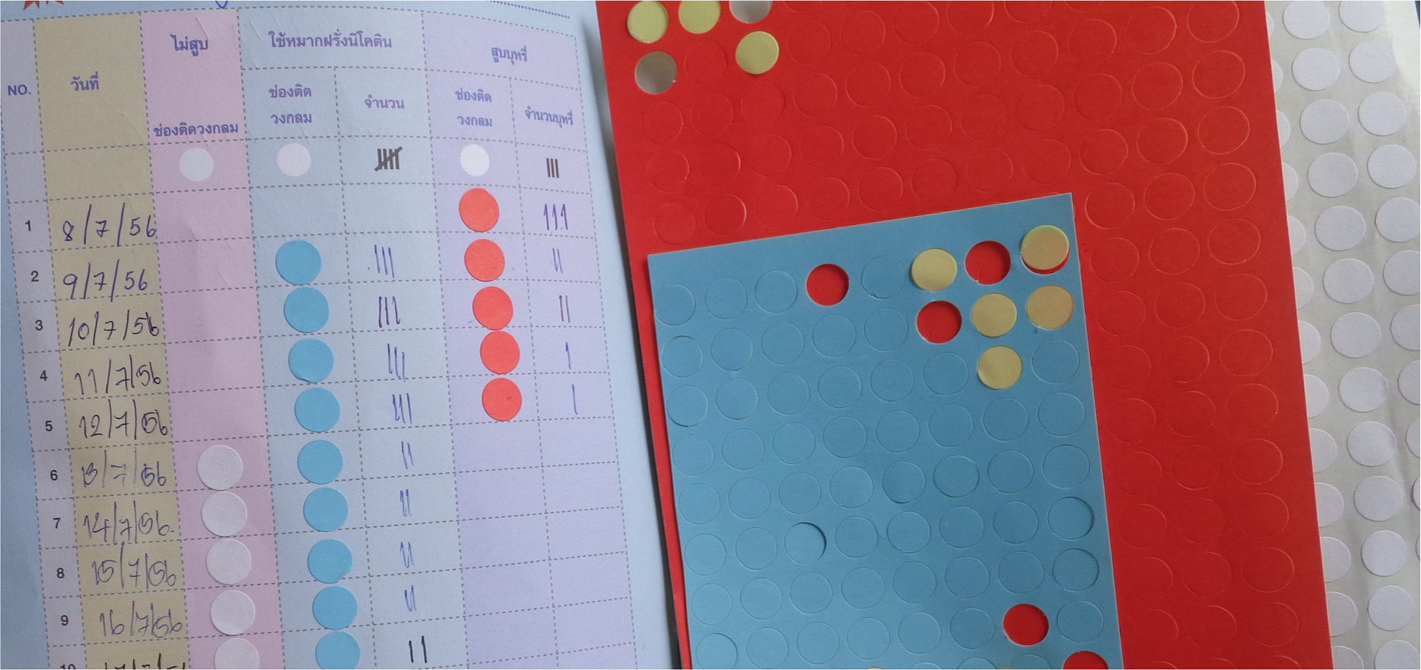
A record book in which the family member will record a smokers’ progress of smoking cessation.

Third, the most striking difference between the two service packages is the assigning, in the new service arm, of a family member to monitor, remind and motivate the smoker until smoking cessation is successful. The family member, trained by the PCU nurse, will be empowered by using an attractive “quit-smoking diary” along with three different colored stickers to record the participant’s choice on smoking each day: smoking (red), NRT chewing gum (blue) and non-smoking (white) (Figure 2). This diary is expected to serve as a reminder to both the smoker and the assisting family member.

Fourth, nicotine replacement chewing gum will be included as one of the components in the new intervention arm. Participants in this arm will receive NRT when they have nicotine withdrawal symptoms [17,18]. A protocol for the provision of NRT has been developed based on Thailand’s Clinical Practice Guideline in Smoking Cessation [19]. NRT will be provided after screening for withdrawal symptoms [17], confirmed by the Fagerstrom test, or breath analysis by smokelyzer or Nicodep test, but only to those willing to use NRT [18,20]. Due to these differences between the two intervention arms, contamination between the two arms is expected to be low.

#### Non-randomized arm (refused to quit smoking)

Once participants have agreed and consented to participate in the trial, they will be asked whether they are willing to attempt to give up smoking (Figure 1). If the participant agrees to give up smoking, he/she will be randomized into the evidence-based new service package arm or the routine service package arm (Figure 1, Table 1). If the participant refuses to attempt to give up smoking, he/she will be automatically allocated to the non-randomized arm. Participants in this arm will not receive any active intervention for smoking cessation, apart from the routine medical service for their existing disease, such as diabetes and hypertension, provided under the universal coverage system.

#### Duration of interventions

The duration of the new smoking cessation service package is as follows:

1. Persistent motivation and coaching by a designated PCU nurse for a period of 3 months.

2. Monthly application of the piCO ^+^ smokerlyzer for a period of 3 months.

3. Assistance by a trained family member (or health care worker in cases of smokers living alone) for a period of 12 months.

The duration of the routine smoking cessation service package is as follows:

1. Counseling of the patient by a healthcare worker during only the first meeting at the NCD clinic of the PCU.

2. Reminding the smoker to stop smoking ad libitum on successive visits for a period of 12 months. The components of each service arm are shown in Table 1.

### Manual and training

An operational manual for the smokerlyzer will be prepared and provided to the study site PCUs. PCU nurses will be trained in how to use smokerlyzers 2 months prior to implementation. Details of the existing smoking cessation service will be reviewed and structured so as to represent the routine service arm in the study. A protocol for nicotine replacement chewing gum will be prepared by the team of researchers, director of Maetha Hospital, physicians, nurses and pharmacists [19]. The indications for NRT will follow the current guidelines for Thailand.

### Definitions

#### Smoking cessation

Smoking cessation is defined as the stopping of smoking for a period of at least 6 months and confirmed by the assessment of carbon monoxide (CO) in the smoker’s expired air using a smokerlyzer.

#### Diabetes

Diabetes mellitus is defined as one of the following criteria: HbA1C concentration of more than 6.5%, or fasting blood glucose of more than 126 mg/dL at least twice, or on the spot blood glucose level of more than 200 mg/dL at least once, simultaneously with 2-h post-prandial blood glucose of more than 200 mg/dL or a history of taking oral hypoglycemic drugs.

#### Hypertension

Hypertension is defined as **(1)** a systolic blood pressure (SBP) of ≥140 mmHg or a diastolic blood pressure (DBP) of ≥90 mmHg in participants who are not taking antihypertensive medication or **(2)** subjects who are taking medication for hypertension.

#### Cardiovascular disease (CVD) events

Myocardial infarction, coronary insufficiency, unstable angina, ischemic stroke, hemorrhagic stroke, transient ischemic attack (TIA), peripheral artery disease or heart failure will be counted as CVD events in this study. Patients with a known diagnosis of CVD will be screened and excluded.

#### CVD mortality

This is defined as any documented death caused by CVD during the study period.

### Study outcome and outcome measurements

#### Primary outcome

The primary outcome of the study is the smoking cessation rate at the 1-year follow-up, proven by measurement of carbon monoxide in parts per million (ppmCO).

#### Secondary outcomes

Secondary outcomes of the trial are:

1. Smoking cessation rate at the 6-month follow-up, proven by measurement of (ppmCO).

2. CVD risk measured by the Framingham general cardiovascular risk score at the 12-month follow-up.

3. Blood pressure and heart rate measured at the 6- and 12-month follow-up.

4. Number of CVD events and deaths at the 12-month follow-up.

5. Cost-effectiveness of the health service packages at the 12-month follow-up.

### Outcome measurements

#### Smoking cessation

This would be assessed by self-reported quitting confirmed by breath analysis of the level of carbon monoxide (CO) in expired air. A piCO ^+^ Smokerlyzer® [21] carbon monoxide monitor will be used for this assessment on the first visit, and again on the 6- and 12-month visits. It is a clinically proven way to determine the carbon monoxide level and easy to use in primary care. In previous studies, the Smokelyzer has been shown to have positive, predictive values for detecting smoking, at more than 95% [12,22]. It can also serve as a motivational tool for giving up smoking. Nicotine dependence will be assessed by the Fagerstrom Nicotine Dependence Test and the Nicodep Test [18,20]. The self-reported smoking cessation rate is ascertained by asking participants the number of cigarettes smoked in the preceding 24 h.

#### Blood pressure

A clinical digital sphygmomanometer will be used for serial measurement of blood pressure.

#### CVD risk assessment

The CVD risk assessment will apply the general Framingham general CVD risk profile for primary healthcare [23,24]. Calculation of the Framingham CVD risk (FMH) score requires information relating to age, sex, blood pressure, diabetes status, smoking status and BMI. The FMH score will be assessed at the baseline, 6- and 12-month visits.

#### Perceived family support

A scale measuring the perceived family support, suitable for the study site setting, was not available yet. Two sections of qualitative inquiries using in-depth interviews among smokers in the Maetha district identified the construct items and afterwards were developed into a scale to measure the smoker’s family support. The scale will be validated in a pilot study of similar population.

#### Health economic assessment

Costs for providing and receiving two intervention services will be estimated by interviewing the health and administrative personnel at MH and the PCUs, as well as the participants. The cost of providing the services includes costs relating to materials, personnel and the facility. The cost of receiving the services includes participants’ time and transportation to the facility. The cost of medical services for CVDs will also be estimated, based on information from the available literature.

#### CVD events and death

Participants will be followed up by nurse-administered, structured questionnaires at the 6- and 12-month visits, ascertaining medical history, lifestyle and health-related behaviors. During each questionnaire, participants will be asked whether they have had a CVD event referred to the community hospital; any reported CVD event will be confirmed by reviewing the NCD network medical records [25].

#### Waist and hip circumference measurement

Waist and hip circumference will be measured at the baseline, 6- and 12-month visits. Stretch-resistant, standard measuring tapes will be provided to nurses to perform the waist circumference and hip circumference measurement. Waist circumference measurement will be made at the approximate midpoint between the lower margin of the last palpable rib and the top of the iliac crest, at the end of normal expiration, according to the WHO protocol [26]. The hip circumference will be measured around the widest portion of the buttocks [26].

### Sample size

A total of 328 participants will be randomized to have 164 in the new service arm and 164 in the routine service arm. The sample size for the randomized controlled trial was calculated to have a power of 80% and 95% confidence interval so as to show the difference in primary and secondary outcomes, and the CVD incidence between the new package arm and the routine service arm. It was calculated using the power sample size calculation of Stata version 11, based on the previous year’s smoking cessation rate at the MH (9%), and the estimated cessation rate of the evidence-based package (24%). Additionally, the calculated sample size was increased by 20% to compensate for loss to follow-up.

The sample size for the additional non-randomized arm was calculated at a ratio of 1:3 compared to the total size of the two intervention arms. The power of 90% and 95 percent confidence interval will suffice to see the difference in CVD events between ex-smokers and continuous smokers, assumed as 0.1 and 0.25. Totally the sample size is 440 participants.

### Data collection and follow-up

Since ESCAPE is a lifestyle modification trial, it requires the measuring of several outcomes and their biological and social covariates. Careful data collection and a well-designed case record form will be applied to avoid the extremes of burden on participants and insufficient data. Training, practice and revision of the case record form will precede the data collection.

#### Baseline survey

The balance between an individual’s motivation to stop smoking and his or her degree of dependence on cigarettes determines successful smoking cessation [27]. Thus, those variables will be measured at the outset of the study and at the end of the intervention. Participants will be asked to provide information about their current and past smoking status, such as the number and type of cigarettes smoked, the time they have an urge to smoke in the morning, the source of their cigarettes and details of previous attempts to give up. Breath analysis by smokerlyzer, the Fagerstrom Test for Nicotine Dependence (FTND) and the Nicodep® Test will be applied in order to assess the participant’s smoking status and nicotine dependence [20]. The smoker’s motivation will be measured by the Motivation to Stop Scale (MTSS), a single-item measure composed of key motivational constructs validated by a previous population-based study [28].

Moreover, this trial is assembled to reveal the impact of smoking cessation on the CVD risk among those most at risk. Thus, the FMH score will be measured at the outset of the study and at the end of the follow-up [23,24]. Blood pressure, heart rate, blood glucose, lipid profile, BMI, and waist and hip circumference will be measured, and these data inputs will be used to provide the FMH score [24]. The patient’s current medication for diabetes or hypertension will be recorded, as well as their food preference (salty food, fatty food) and their family history of smoking, including any known CVD events or deaths.

The new evidence-based service intervention will focus not only on the individual, but also on the social environment of the individual, such as their family support. Thus, perceived family support will be measured at the outset. Demographic and socioeconomic information of the participants will also be collected, as well as information regarding costs relating to travel, the provider, client and health service. As a covariate, individual health literacy will briefly be assessed. Measurement of this latent variable will apply communicative and critical health literacy assessment, developed and validated by Ishikawa et al. [29].

#### Follow-up

The study outcome measurement and follow-up schedule is detailed in Table 2. Several ideas to limit the missing data were followed in designing the ESCAPE trial [30]. This trial will be undertaken following the routine schedule of the NCD clinics to which diabetes and hypertension patients make regular visits for medical checkups and receiving drugs. Since the study population is routinely served by the PCUs, the chance of dropout is limited. Participants are regular and long-term clients of PCU health services. The rapport between NCD patients and PCU nurses may strengthen the follow-up and minimize the dropout in the trial.

**Table 2:** Overview of data collection and measurements in all three arms of ESCAPE study

	**Follow-up**												
Visit number	1	2	3	4	5	6	7	8	9	10	11	12	13
Month	0	1	2	3	4	5	6	7	8	9	10	11	12
Inclusion and exclusion criteria	x												
Framingham scoring	x						x						x
Self-reported smoking cessation	x			x			x						x
Smokerlyzer	x						x						x
Fagerstrom Test	x						x						x
Blood pressure	x	x	x	x			x			x			x
BMI	x			x			x						x
Blood glucose	x						x						x
Waist circumference	x						x						x
Hip circumference	x						x						x
HbA1C	x												
Lipid profile	x												
CVD event	x						x						x
CVD death	x						x						x
Questionnaires													
Demography	x												
Questionnaire assessing motivation to stop smoking (MTSS)	x						x						x
Questionnaire assessing cost effectiveness	x						x						x
Questionnaire assessing family support on smoking cessation	x						x						x
Questionnaire assessing health literacy	x						x						x

Moreover, the intervention design is an add-on design in which additional evidence-based components are added to the existing routine service [30]. Regarding the outcome measurement, most of the secondary outcomes are feasible to measure via the routine data collection of the study site hospital network. Furthermore, the sample size has been increased by 20% to compensate for dropouts.

The study is carefully designed randomized controlled trial, evaluating two approaches of active intervention and comparing the rate of behavior change. However, the CVD outcome event rate of the active intervention arms will also be analyzed and compared with that of the non-randomized arm made up of smokers refusing to try to give up smoking. It will require the adjustment of minimal residual confounders. Moreover, there will be different models of covariates used to analyze different outcomes. For instance, adjustments in the comparison of CVD events and deaths between the intervention and control arms may include family history, lifestyle, physical activity, alcohol consumption, dietary habits, current medications and socioeconomic status [25]. To assess physical activity as a covariate, a rapid physical activity assessment scale will be applied [31]. Therefore, possible confounders are collected and will be adjusted appropriately in later analysis [32] (Table 3).

**Table 3:** Confounder measurements

**Confounder**	**Variables**	**How to measure**
Demographic characteristics	Age, sex, social status, family size, educational attainment, occupational history, income	Questionnaire
Lifestyle	Alcohol consumption, physical activity	Questionnaire
Genetic factor	Family history of diabetes, hypertension and CVD	Questionnaire
Drugs	Different medications for diabetes and hypertension	Clinical record
Health literacy [29]	A scale of five questions	Questionnaire of Ishikawa’s scale [29]
Family support	A scale of 16 questions	Questionnaire
Physical activity	RAPA scale [30]	Questionnaire

The level of perceived family support and its impact will vary among participants. Participants in the routine service arm and the refusing to give up arm are not supposed to receive care by a family member, purposefully and systemically trained and empowered by the nurse. However, a participant in the control arm may, by chance, also receive strong family support. In view of this major difference, the impact of family support would be measured and adjusted statistically via multivariable analysis.

### Analyses plan

All analyses for pre-specified outcomes in the trial protocol will strictly follow the intention-to-treat principle [33]. Statistical significance in all statistical analysis will be taken as a *P* value less than 0.05 with a 95% confidence interval. Relative risk (RR) for achieving primary outcome, smokerlyzer-confirmed smoking cessation, will be reported with the 95% confidence interval. Moreover, the number needed to treat (NNT) for achieving smokerlyzer-confirmed smoking cessation by the two intervention arms will be reported [34]. To overcome the contamination of the intervention, complier average causal effect (CACE) analysis will necessarily be applied.

#### Smoking cessation rate

The Smokerlyzer-confirmed smoking cessation rate will be compared between two arms of the RCT, at 6 months and 12 months of follow-up, by applying the chi-square test. The smoking cessation rate will also be compared among the three arms of the study. The factors influencing successful smoking cessation at 12 months will be analyzed by multivariable regression model-adjusting covariates. Continuous variables, such as expired CO levels, the Fagerstrom score and nicotine dependence levels, will be compared using MANOVA.

#### CVD risk, CVD events and CVD death

CVD risk via FMH scores, blood pressure, heart rate and BMI will be compared between those successfully giving up smoking and continuous smokers by adjusting covariates. Moreover, the time event analysis approach will be applied to compare the CVD events and CVD deaths between the two intervention arms. Those outcomes will then be analyzed among the three arms of the study by applying the Cox regression model.

#### A path model of smoking cessation behavioral change

**Figure 3:**
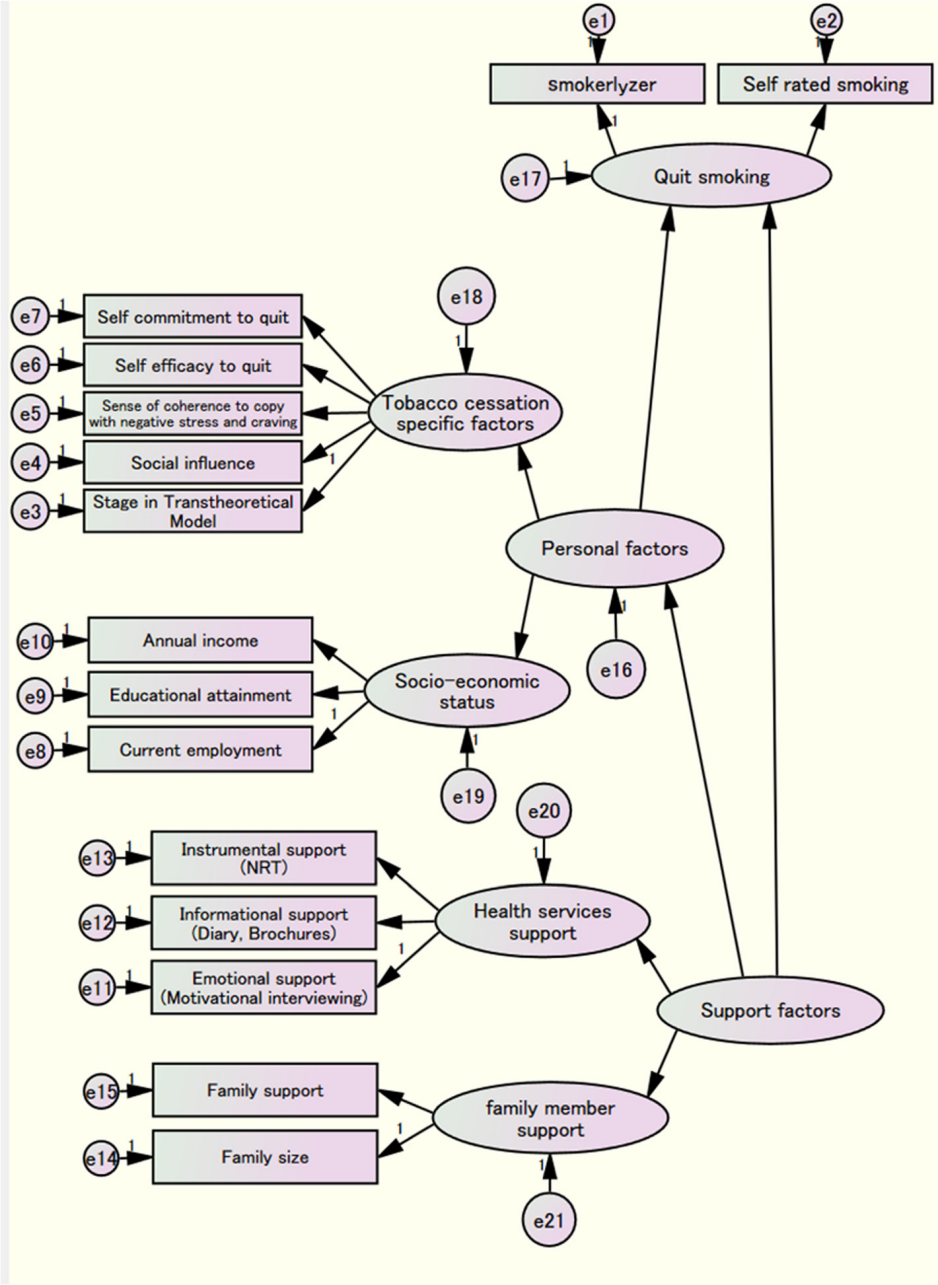
A path model of the smoking cessation behavior change hypothesis.

The structure equation model will be used to determine the factors influencing the behavioral change of patients at risk of CVD to stop smoking (Figure 3). Individual factors, such as age, sex, motivation to stop smoking and health literacy; social factors, such as perceived family support, income, household smoking prevalence and residence address; health service factors, such as medications and NRT use; tobacco-related factors, such as the number of cigarettes smoked, tobacco cost and cigarette type; and disease conditions, such as hypertension, BMI, diabetes and CVD risk on smoking cessation, will be analyzed using a holistic approach.

#### Cost-effectiveness analysis

The cost of interventions will be compared in terms of outcome achievement. The additional cost of producing an additional percentage of successful quitters at 1-year follow-up will be calculated. Based on the Framingham general cardiovascular risk score, differences in CVD events and deaths and the associated costs between the arms will be estimated for a period of 10 years at the end of the 1-year follow-up. Lifetime costs and health outcomes for our study population (aged 35 years and above) will be estimated using data both in and outside Thailand. Outcomes of the analysis will be: CVD events and deaths averted and cost over 10 years; incremental cost-effectiveness ratio (ICER) per CVD death averted; lifetime costs and quality-adjusted life years (QALYs) gained; and ICER per QALYs gained. The analysis will be performed using both the provider and societal perspective. All the future costs and outcomes will be discounted at a rate of 3% per annum. Sensitivity analysis will be employed to check the robustness of the results.

#### Qualitative analysis

The characteristics of a participant successfully giving up smoking will be analyzed qualitatively as a positive deviance.

## Discussion

Smoking cessation is considered to be the most urgent and high-priority intervention for prevention of non-communicable disease crisis internationally [1]. WHO estimated that 17.3 million people die of CVDs each year, 80% of which take place in low- and middle-income countries [4,10]. CVDs are preventable, and a substantial number of CVD-related deaths can be prevented by smoking cessation. Thailand, a middle-income country, is witnessing a rise in smoking prevalence, especially among the male population, with significant health and economic impacts [5,35,36]. Therefore, despite the nationwide controls on tobacco, strengthening the smoking cessation health service is an urgent necessity in Thailand [35]. The ESCAPE study aims to fill this gap by devising a multicomponent smoking-cessation service applicable at the primary care level.

**Figure 4:**
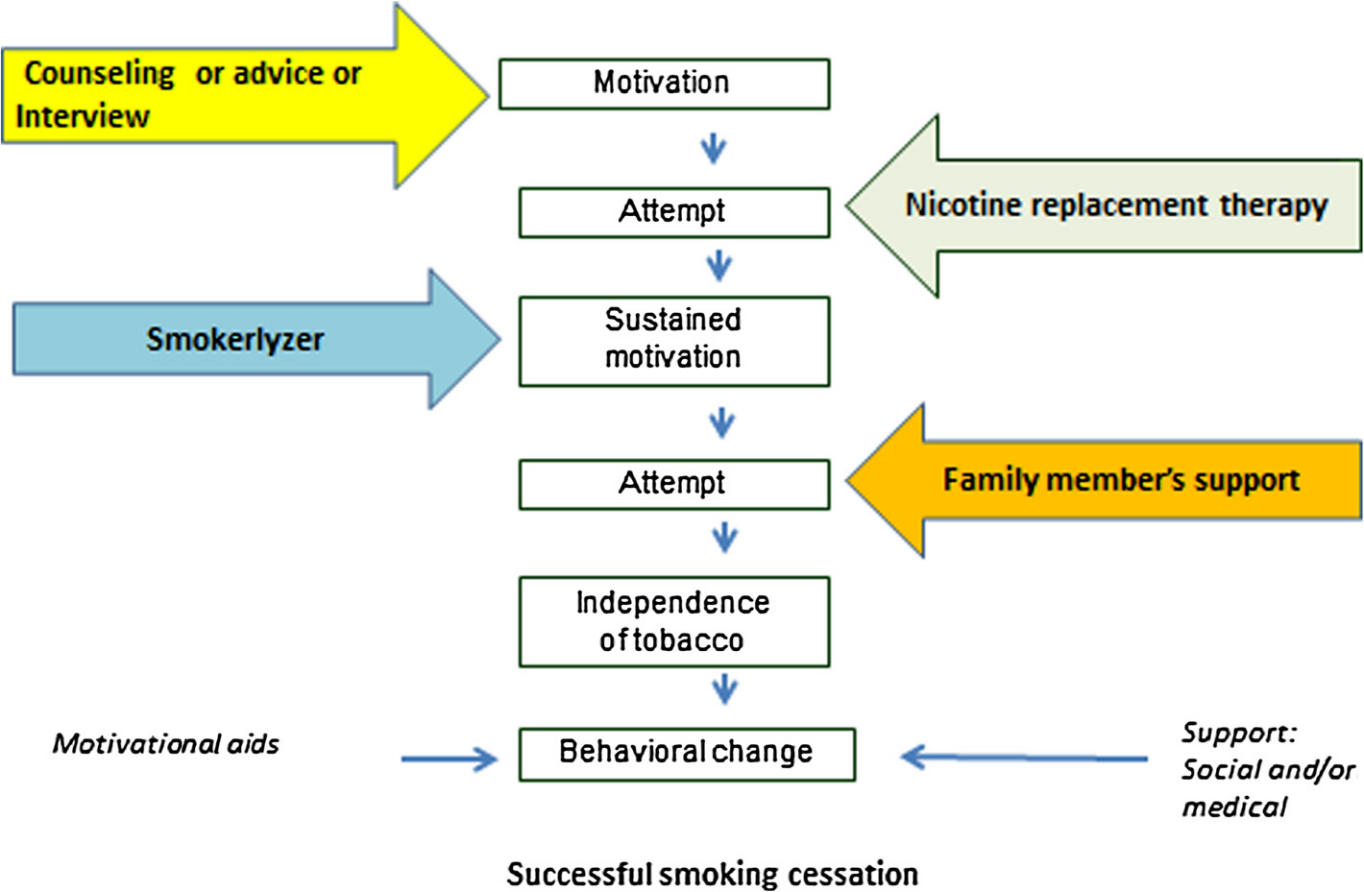
A conceptual model of evidence-based interventions and their actions in the development of behavioral change to stop smoking.

In considering the individualized needs of patients in their attempts to give up smoking, a care approach package has been designed, the steps of which are outlined in Figure 4.

Existing evidence shows that interventions focusing on the motivation to give up smoking, such as motivational interviewing or brief counseling, can increase the rate of smoking cessation [37]. Having generated initial motivation to give up smoking, the challenge, in practice, is to sustain this motivation [27,38] (Figure 4). Evidence consistently states that the persistent offering of assistance, medication or behavioral support with the aim of smoking cessation is more effective than brief advice or a single session of counseling [38]. Recently, the sustainable impact of the nurse’s or allied healthcare worker’s empirical assertive approach was reported to have had a positive effect on cardiovascular risk factors, including smoking cessation [16]. Hence, this approach will be used in the current study for the new service arm.

Nursing interventions increase the smoking cessation rate, but the monitoring of smoking behavior remains a challenge [37]. In this study, PCU nurses will use smokerlyzers in the new service arm to monitor smokers’ attempts to give up smoking. Breath analysis results will be shown to participants, enabling them to see the decline in the CO level of their expired air.

Pharmacological interventions, such as nicotine replacement therapy (NRT), have been shown to work effectively within developed settings [39]. NRT relieves nicotine withdrawal symptoms and thus helps to sustain motivation to stop smoking [40]. However, in the developing setting of Thailand, while NRT has passed safety tests [41], the medication is not yet available via the publically financed health service system. Moreover, recent studies in Thailand heterogeneously reported that smokers and healthcare providers resorted to locally available herbs and remedies to relieve nicotine withdrawal and tobacco craving symptoms [12,42]. Thus, another challenge in designing an effective smoking cessation service is to provide free medication at the primary health care level to relieve nicotine withdrawal symptoms. Nicotine replacement chewing gum, therefore, will be included as one of the components in the new intervention arm of the ESCAPE study. Its cost-effectiveness will be analyzed with a view to replication in similar developing country settings.

Next, the need for support and care may differ among smokers (Figure 4); some patients may need motivation, some may need relief from craving symptoms by nicotine replacement therapy, and some may need buddy support [38]. Close monitoring and the kind support provided by a family member help smokers to succeed in giving up smoking [38,43]. Moreover, public health intervention aiming at lifestyle changes needs to focus on individual as well as social and environmental influences. In consideration of this, a component will be added to the new service package, that of close monitoring and continual support of the smoker by a family member (or other assigned member) using a sticker record diary. Social support may empower the smoker in his/her attempts to give up, while relieving the stigma of smoking that may be experienced by other family members [43].

Existing evidence suggests that a smoking cessation service, delivered in combination with other health services, might be a more effective approach than the conventional delivery through tobacco clinics [44,45]. The ESCAPE study is strategically designed to improve access to a smoking cessation service within the community. In this study, NCD clinics, regularly attended by patients at risk of CVD, are chosen as the centers to provide the smoking cessation service. Thus, smokers attempting to stop smoking will have easy access to the health service within their community. Moreover, this approach is expected to strengthen the follow-up and minimize the dropout number of participants during the research.

In this study a smoking cessation intervention at the primary health care level in the setting of northern Thailand will be tested. The 5 A approach is currently adopted by many hospitals in Thailand to treat smokers. The existing systems used at the study sites and the human resources available were observed. Based on existing global evidence, a new smoking cessation intervention package has been designed to treat smokers at the primary health care level. Its efficacy will be assessed by outcomes of behavioral change and cardiovascular risk reduction, and the cost effectiveness of achieving those outcomes. It is expected to generate a culturally tailored, practically effective smoking cessation service replicable in other developing countries.

## Trial status

Recruiting.

## Abbreviations

RCT: Randomized controlled trial; MH: Maetha hospital; PCU: Primary health care unit; FMH score: General Framingham scoring; CVD: Cardiovascular disease; SEM: Structural equation modeling; ICER: Incremental cost-effectiveness ratio; QALY: Quality-adjusted life years.

## Competing interests

There are none to declare. The investigators did not receive funds from any pharmaceutical or equipment company.

## Authors’ contributions

MNA and MY were the lead authors in charge of study design. All authors worked with MNA to design the interventions, revise and finalize the study protocol. MY made the path model for smoking cessation. HF and HY provided the advice to set the outcome measures and devise the diary. SM provided the random sequence generation and block randomization format as well as the ethics application. TK provided the health economic assessment. KM provided the hip and waist measurements. ST provided the collection of spatial epidemiological information. SC and YS advised MNA in designing the family support component; TL, PN and PT provided the measure for family support as well as the ethics application in Thailand. YH, KO, SN, SK and EM also provided advice and comments for this study. EM and MY provided guidance to MNA on the analysis plan. EM, MY and MNA registered the trial. MNA wrote the manuscript and finalized the article. All authors read and approved the final manuscript.
